# Sprained Ankles

**Published:** 1894-12

**Authors:** 


SPRAINED ANKLES.—From time to time, one hears of different means of caring for sprained ankles, turned ankles, twisted wrists, etc., but the way now in vogue seems to give better results than any in the past.

It is generally within an hour after the accident that you are called in to see the case. The patient is suffering vrry severely, and wanting very much to know if "anything is broken." After examining foı fractures, order the part to be bathed in *extremely hot* water, every hour or two, for a period of fifteen minutes at a time. Have the water just as hot as the patient can bear it, and apply with a sponge or cloth, rather than allow the ankle to lie in the water. Then dry and let the part rest quietly, wrapped in flannels, when an application of hamamelis, or veratrum and hamamelis, may be made.

Before retiring, apply a flannel bandage tightly around the swollen part, only being careful that the circulation is not shut off.

It is surprising how the hot applications relieve the pain and produce absorption, and how the bandage, by pressure, prevents swelling and inflammation.

